# Development of a highly active engineered PETase enzyme for polyester degradation

**DOI:** 10.1111/febs.70228

**Published:** 2025-08-23

**Authors:** Shapla Bhattacharya, Rossella Castagna, Hajar Estiri, Toms Upmanis, Andrea Ricci, Alfonso Gautieri, Emilio Parisini

**Affiliations:** ^1^ Department of Biotechnology Latvian Institute of Organic Synthesis Riga Latvia; ^2^ Institute of Chemistry and Chemical Technology, Faculty of Natural Sciences and Technology Riga Technical University Latvia; ^3^ Dipartimento di Chimica Materiali e Ingegneria Chimica “G. Natta”, Politecnico di Milano Milan Italy; ^4^ Faculty of Chemistry University of Latvia Riga Latvia; ^5^ Biomolecular Engineering Lab, Dipartimento di Elettronica, Informazione e Bioingegneria Politecnico di Milano Milan Italy; ^6^ Department of Chemistry “G. Ciamician” University of Bologna Italy

**Keywords:** enzymatic depolymerization, PETase, plastic degradation, rational protein engineering, thermal stability

## Abstract

Polyethylene terephthalate (PET) accounts for ≈6% of global plastic production, contributing considerably to the global solid‐waste stream and environmental plastic pollution. Since the discovery of PET‐depolymerizing enzymes, enzymatic PET recycling has been regarded as a promising method for plastic disposal, particularly in the context of a circular economy strategy. However, because the PET‐degrading enzymes developed so far suffer from relatively limited thermostability and low catalytic efficiency, as well as degradation product inhibition, their large‐scale industrial applications are still largely hampered. To overcome these limitations, we engineered the current PET‐hydrolyzing enzyme gold standard [the ICCG variant of leaf‐branch compost cutinase (LCC‐ICCG)] using *in silico* protein design methods to develop a PET‐hydrolyzing enzyme that features enhanced thermal stability and PET depolymerization activity. Our mutant, LCC‐ICCG‐C09, features a 3.5 °C increase in melting temperature relative to the LCC‐ICCG enzyme. Under optimal reaction conditions (68 °C), the engineered enzyme hydrolyzes amorphous PET material into terephthalic acid (TPA) with a two‐fold higher efficiency compared to LCC‐ICCG. Owing to its enhanced properties, LCC‐ICCG‐C09 may be a promising candidate for future applications in industrial PET recycling processes.

## Graphical abstract

Polyethylene terephthalate (PET) accounts for approximately 6% of global plastic production and is a major contributor to plastic pollution. Enzymatic recycling offers a promising solution, but current PET‐degrading enzymes often lack sufficient thermostability and catalytic efficiency. Through *in silico* design, we engineered a more robust variant, LCC‐ICCG‐C09, which exhibits enhanced thermal stability and twice the depolymerization efficiency of its predecessor, LCC‐ICCG. This mutant shows strong potential for industrial‐scale PET recycling applications.

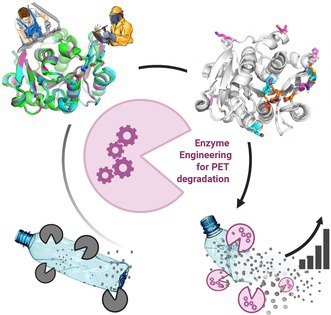

**Abbreviations**
BHET: bis(hydroxyethyl)terephthalate
CD: circular dichroism
DSF: differential scanning fluorimetry
EG: ethylene glycol
HPLC: high‐performance liquid chromatography
IPTG: isopropyl 1thioβD galactopyranoside
LB: Luria–Bertani broth
LCC: leaf‐branch compost cutinase
MD: molecular dynamics
MeCN: acetonitrile
MHET: mono(hydroxyethyl)terephthalate
PDB: Protein Data Bank
PET: polyethylene terephthalate
PMSF: phenylmethylsulfonyl fluoride
pNPA: p‐nitrophenyl acetate
RMSD: root mean square deviations
*T*_m_
: melting temperature
TPA: terephthalic acid

## Introduction

Worldwide plastic production reached 390 million metric tons in 2021, of which 90.2% comes from fossil‐based production and only the remaining 9.8% comes from post‐consumer plastic recycling or bio‐based plastics [1]. This massive production and usage of plastics, along with their very long persistence in the environment, has led to an extreme global pollution threat, especially in marine environments [2, 3, 4, 5].

Polyethylene terephthalate (PET), the most abundant synthetic polyester in the environment, has an average lifetime of 25–50 years and accounts for 6.2% of the total plastic production [1]. It is widely used in the production of textile fibers and resins for single‐use beverage bottles and packaging. Polyethylene terephthalate is a thermoplastic polymer that is composed of terephthalic acid (TPA) and ethylene glycol (EG) subunits. Nowadays, PET is, to a large extent, mechanically recycled, a process that results in a significant loss of the material's properties and value [6]. Harsh chemical treatments involving the use of sulfuric acid at 150 °C or carried out under alkaline conditions in the presence of hazardous chemical catalysts (e.g., methyltrioctylammonium bromide) are also used, leading to the depolymerization of PET into its monomeric building blocks via ester bonds cleavage. However, the use of such harsh conditions makes it problematic to expand this treatment on a large scale.

In 1977, Tokiwa and Suzuki proposed using lipase enzymes to degrade polymeric materials [7]. Indeed, enzymes work in mild conditions and can replace hazardous chemicals, a concept known as green chemistry. The first report of an efficient PET hydrolase (from *Thermobifida fusca*) was published in 2005 [8]. Since then, numerous thermally stable PET hydrolases and their related enzymes from the cutinase group (EC. 3.1.1.74) have been identified in different organisms [9, 10, 11, 12, 13]. The search for thermostable PET hydrolases is driven by the fact that PET can be more effectively hydrolyzed at temperatures close to its glass transition temperature (≈70–80 °C in air, ≈60–70 °C in water). Near the glass transition temperature, the polymer chains become more flexible, enabling PET hydrolases to function optimally. In 2016, Yoshida *et al*. [14] reported on a mesophilic bacterium (*Ideonella sakaiensis*) that can thrive on an amorphous PET film as its primary carbon source already at 30 °C, making the enzyme responsible for PET hydrolysis (*Is*PETase) the best option for PET waste decomposition. This work spurred a great deal of interest and several efforts in providing enhanced *Is*PETase mutants. However, since *Is*PETase is heat‐labile, the enzyme quickly loses its activity at temperatures above 40 °C. Therefore, more suitable scaffolds have also been explored, such as, for instance, the Leaf‐branch Compost Cutinase (LCC), a naturally occurring PETase that has been reported to outperform all other known PET‐degrading enzymes and to present a melting temperature (*T*_*m*_) of 84.7 °C. This enzyme has been noticeably engineered in 2020 by Tournier *et al*. [15] leading to the so‐called ICCG variant (also known as LCC‐ICCG, or ICCG for brevity), which, in two different accounts, is reported to feature a *T*_*m*_ of 91.7 or 94.0 °C (Table 1).

**Table 1:** Relevant PETase enzymes reported in the literature.

Parent enzyme	Name	Year	Tm (°C)	Ref.
*Is*PETase	(wild type)	2016	46.0	Yoshida *et al*. [14]
*Is*PETase	IsPETaseTM	2019	57.6	Son *et al*. [44]
LCC	(wild type)	2020	84.7	Tournier *et al*. [15]
LCC	LCC‐ICCG	2020	94.0	Tournier *et al*. [15]
LCC	LCC‐ICCG	2023	91.7	Arnal *et al*. [28]
*Is*PETase	—	2021	61.2	Meng *et al*. [45]
*Is*PETase	IsPETaseTM^K95N/F201I^	2021	61.6	Brott *et al*. [46]
*Is*PETase	DuraPETase	2021	77.0	Cui *et al*. [47]
*Is*PETase	HotPETase	2022	82.5	Bell *et al*. [25]
PES‐H1	L92F/Q94F	2022	86.7	Pfaff *et al*. [48]
*Is*PETase	FastPETase	2023	67.1	Lu *et al*. [49]
*Is*PETase	DepoPETase	2023	69.4	Shi *et al*. [50]

To the best of our knowledge, this variant is currently considered to be the gold standard PETase enzyme [16]. Despite its remarkable stability, the sequence space for even a small enzyme like LCC is astounding (20^293^ or 10^381^ theoretical possible sequences), leaving room to search for even more stable and active enzymes. Here, we set out to design improved PETase enzymes starting from the LCC‐ICCG variant and using design strategies similar to those that we used previously with other classes of enzymes [17, 18, 19, 20]. Our working hypothesis is that improving the thermal stability would be beneficial for the PETase enzymatic activity, as observed in notable examples (IsPETase < LCC < LCC‐ICCG). For this reason, we set out to improve LCC‐ICCG thermal stability, expecting further beneficial effects such as, for instance, higher expression yield and enhanced enzymatic activity.

The field of computational design of thermostable proteins is relatively mature, with several established available methods that can help to generate a library of mutants and rank them. The easiest and more common approach involves the introduction of one or more disulfide bonds, which can rigidify the backbone of the protein and make it more stable. Other stabilization methods generally aim at extending the intramolecular hydrogen bonding network, creating intermolecular salt‐bridges, or providing better packing of the hydrophobic core (e.g., PROSS) [21]. The Rosetta Supercharge method is meant to improve the stability of the protein by making the surface more hydrophilic, a feature that is usually associated with more stable proteins [22].

While these methods are fast and have been successfully used, most of them use an implicit solvent model (i.e., the solvent is modeled with a continuum rather than with explicit atoms) and assume a rigid backbone (which may limit the long‐range structural effects of a mutation). To overcome these limitations, we included a further computational filter based on Molecular Dynamics simulations in explicit water. By using an orthogonal method, we aimed at screening and ranking on a common platform the most promising mutants generated by the methods described above.

Our efforts led to the development of three variants that feature *T*_*m*_ values higher than current PETases and, for one of them (LCC‐ICCG‐C09), further enhanced catalytic activity on amorphous PET films.

## Results

### *In silico* design and selection of stabilizing mutants

Enzyme engineering was initiated using the LCC‐ICCG variant reported by Tournier *et al*. [15], a mutant of the wild‐type leaf‐branch compost cutinase (LCC) bearing four point substitutions: F243I, Y127G, S283C, and D238C. The latter two substitutions introduce a second disulfide bond, complementing the native C275–C292 bond. In both wild‐type and mutant enzymes, the catalytic site is centered on residue S165. The crystal structure of the inactive S165A LCC‐ICCG variant is available in the Protein Data Bank (PDB ID: 6THS). Our design strategy followed established protocols [17, 18, 19] beginning with the reversion of residue 165 to the catalytically active serine. Residues within 5 Å of S165 were excluded from mutagenesis to preserve active site integrity. Three distinct computational methods were employed, yielding three separate series of variants.

The use of the Rosetta Supercharge method [22] which aimed at increasing the charge on the surface of the enzyme, led to the identification of 1 000 potential enzyme variants, from which those 10 that featured the best Rosetta score (which we named C01‐C10) were selected for further analysis. PROSS design [21] produced 9 variants (P01‐P09), and Disulfide‐by‐Design [23] identified 125 enzyme variants, from which we selected the best 10, named X01‐X10.

**Fig. 1:**
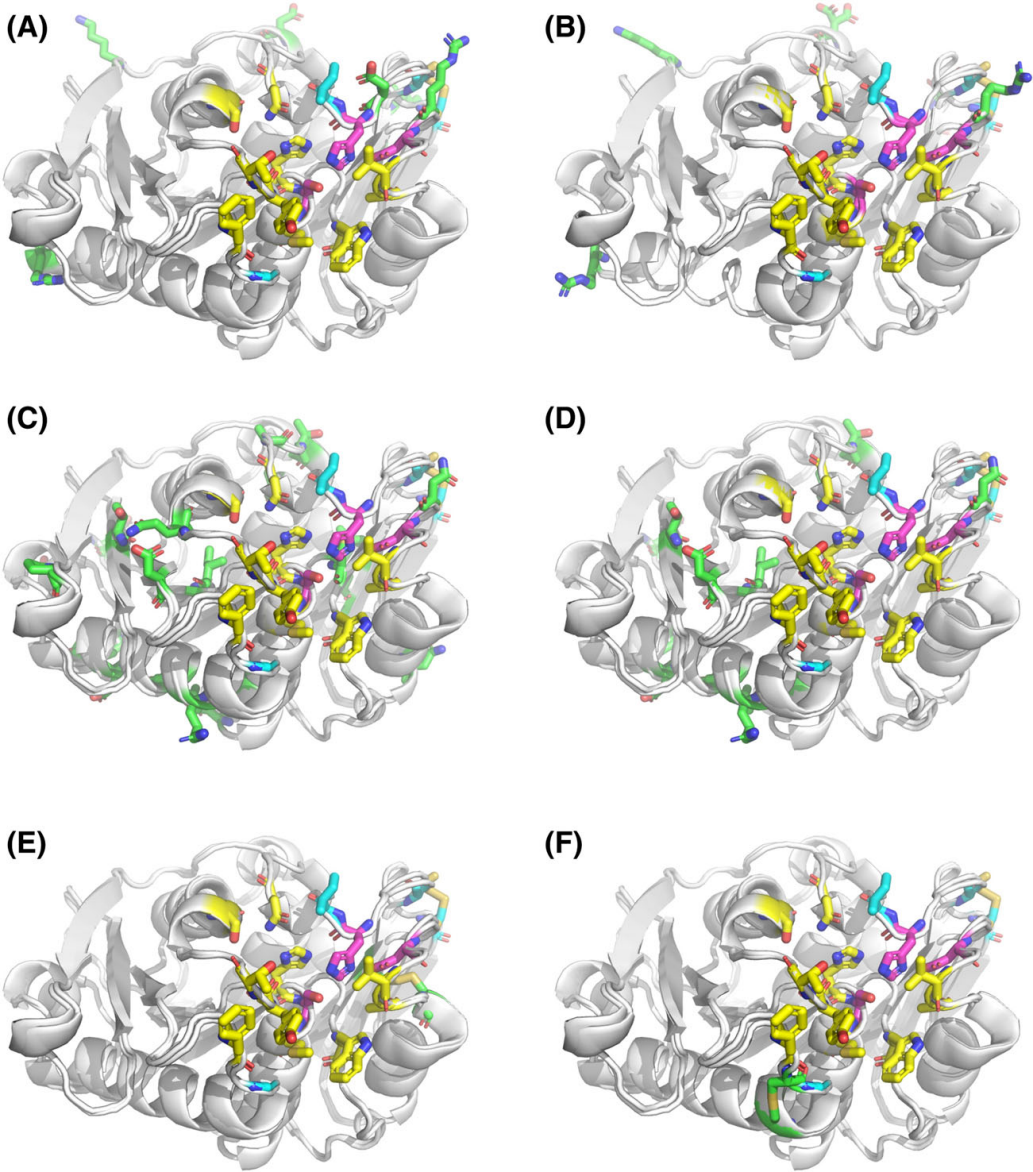
Structural comparison of the enzyme variants selected for experimental characterization. Pink sticks represent the catalytic triad, cyan sticks represent the four substitutions introduced in LCC‐ICCG, and green sticks represent additional substitutions introduced in each new variant (A: C08, B: C09, C: P06, D: P08, E: X05, F:X09). Figures are generated with the software PyMol [51].

The 29 candidate enzyme variants originating from the different computational screening methods were then subject to 1 μs molecular dynamics (MD) simulations in explicit water and ranked based on their stability as assessed by RMSF analysis [17]. Our MD‐based ranking led to the selection of six candidate mutants (C08, C09, P06, P08, X05, X09) for experimental production and characterization (Fig. 1, Fig. S1 and Table 2).

**Table 2:** List of substitutions relative to the sequence of the wild‐type LCC enzyme. In bold are the substitutions already present in the LCC‐ICCG scaffold.

Enzyme	Number of substitutions	Substitutions
LCC‐ICCG [15]	4	Y127G, D238C, F243I, S283C
C08	11	S48D, S57K, **Y127G**, S145R, A209R, **D238C**, S241D, **F243I**, N276D, N278K, **S283C**
C09	13	S36D, Q40R, S48D, S57K, **Y127G**, S145R, A209R, **D238C**, **F243I**, N276D, N278K, **S283C**, Q293K
P06	16	V75T, L84E, M91I, N120D, **Y127G**, S133R, S136L, N140D, A156P, I174A, A209N, **D238C**, **F243I**, A250T, S253A, **S283C**
P08	26	S64P, V75T, L84E, M91I, A99Q, N120D, **Y127G**, S133R, S136L, N140D, R143V, A152S, A156P, I174A, N178D, T188C, A209N, Q224N, V233M, V235I, **D238C**, **F243I**, N248S, A250T, S253A, **S283C**
X05	6	**Y127G**, G206C, V215C, **D238C**, **F243I**, **S283C**
X09	6	D126C, **Y127G**, S130C, **D238C**, **F243I**, **S283C**

### Thermal stability

The six candidate enzymes were expressed in *E. coli* and purified. The final yields of all the enzymes were around 16 mg·L^−1^ of bacterial culture (see yield in Table S1). Wild‐type LCC, a naturally occurring PETase, has a *T*_*m*_ of 84.7 °C. This enzyme has been previously engineered by Tournier *et al*. [15] leading to the LCC‐ICCG variant with a reported *T*_*m*_ of 94.0 °C (93.6 °C in our own assessment, a difference that could be attributed to that fact that, for *T*_*m*_ measurements, Tournier *et al*. used differential scanning fluorimetry (DSF), whereas we used circular dichroism (CD)). Indeed, during the thermal stability assessment of the enzymes, it became apparent that the determination of its melting temperature (*T*_*m*_) via differential scanning fluorimetry (DSF) was unreliable. Specifically, the denaturation curve could not be accurately derived due to the *T*_*m*_ approaching 100 °C, which is the upper operational limit of the Applied Biosystems 7500 Real‐Time PCR System utilized for the DSF measurements. As a result, a complete unfolding transition (typically indicated by a plateau) could not be observed for the enzymes within the instrumental constraints. To overcome this limitation and enable a consistent comparison across all enzyme variants, we employed CD spectroscopy. This technique facilitated the accurate determination of *T*_*m*_ values for all proteins examined in the study. Consequently, we propose that the discrepancy in the *T*_*m*_ of ICCG reported by Tournier *et al*. compared to our findings likely arises from the methodological differences between DSF and CD‐based measurements.

After our enzyme design campaign, we tested the thermal stability of the six engineered variants (Table 3); we found that the C09 (*T*_*m*_ = 97.1 °C) and X05 (*T*_*m*_ = 96.9 °C) variants have a higher *T*_*m*_ than the reference LCC‐ICCG (*T*_*m*_ = 93.6 °C). The X09 (*T*_*m*_ = 93.8 °C) variant exhibits similar thermal stability to LCC‐ICCG (Fig. S2). We did not determine the melting temperature of P06 and P08 due to their negligible enzymatic activity in preliminary experiments, while for C08 it was not possible to obtain a reliable fit.

**Table 3:** Assessed melting temperature for different PETases and improvement with respect to the LCC wild‐type enzyme. The estimation of *T*_*m*_ is sensitive to the experimental setup and conditions, which could explain the difference between the reported *T*_*m*_ and the *T*_*m*_ determined by us. For proper comparison, we assessed the *T*_*m*_ of LCC‐ICCG using the same setup that we used for the measurement of the *T*_*m*_ of our engineered enzymes. The melting temperature was not measured for P06 and P08, while for C08 it was not possible to obtain a reliable fit. n.d., not determinable; n.m., not measured.

Enzyme	*T*_*m*_ (°C)	Δ*T*_*m*_ (°C)	References
WT‐LCC	84.7	–	Tournier *et al*. [15]; Pirillo *et al*. [24]
LCC‐ICCG	94.0	+9.3	Tournier *et al*. [15]
LCC‐ICCG	91.7	+7.0	Arnal *et al*. [17]
LCC‐ICCG^a^	93.6 ± 2.1	+8.9	
C09	97.1 ± 0.8	+12.4	
X05	96.9 ± 2.9	+12.2	
X09	93.8 ± 1.0	+9.1	
C08	n.d.		
P06	n.m.		
P08	n.m.		

^a^ Reassessed in this work.

### Enzymatic activity

**Fig. 2:**
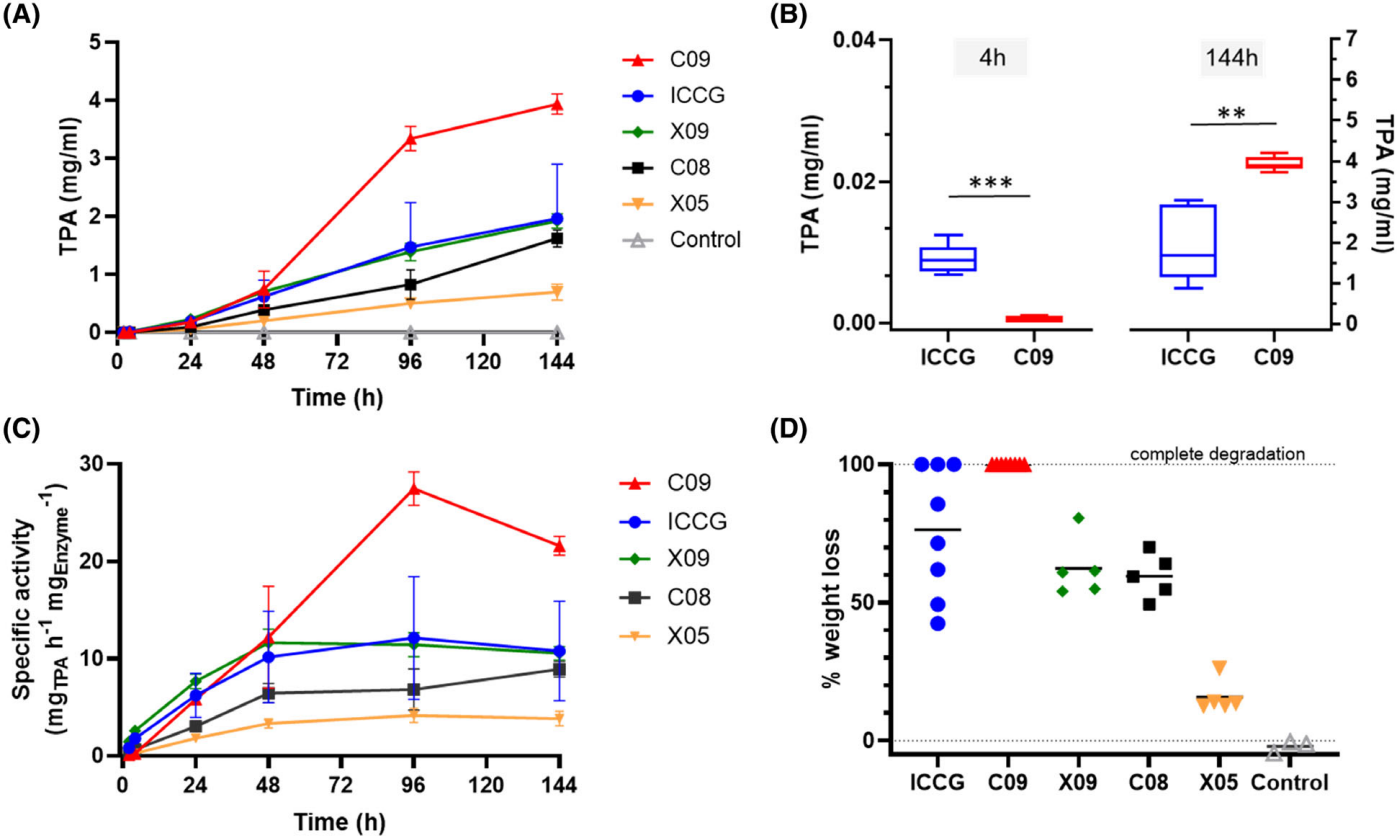
Comparison of polyethylene terephthalate (PET) depolymerization over time for all enzymes. (A) Terephthalic acid (TPA) production over time at 68 °C (LCC‐ICCG – C09 – X05 – X09 – C08 40 nm, pH 8.0). Means ± standard deviations (*n* = 5) are shown. Data points have been reused in Figs S3A and S5. (B) Comparison of TPA production at 68 °C at 4 h and at 144 h. **P < 0.01; ***P < 0.001 (one‐tailed unpaired Welch's *t*‐test). Means ± standard deviations (*n* = 5) are shown. Data points have been reused in Fig. S5. (C) Enzyme‐specific activity at the different time points (LCC‐ICCG – C09 – X05 – X09 – C08 40 nm, 68 °C, pH 8.0). Means ± standard deviation (*n* = 5) are shown. Data points at 144 h have been reused in Fig. S6B. (D) Percentage of PET film weight loss after treatment (6 days at 68 °C, LCC‐ICCG and C09 *n* = 8, X09 – C08 –X05 *n* = 5, control *n* = 3). The mean % of weight loss (black bar) for LCC‐ICCG at day 6 is 76%, while it is 100% for C09.

Terephthalic acid (TPA) is one of the major degradation products of the activity of the enzymes on PET. This compound can be detected by liquid chromatography (HPLC), which allows a precise quantification of product formation. Given the potential absorption spectra overlaps of the degradation products that will affect the absorption spectra recording in spectrophotometric methods, we chose HPLC for its higher specificity and precision in quantifying the enzymatic reaction products as also described in the literature [24]. To assess enzymatic activity, aliquots of the enzymatic reactions were harvested at multiple time points and analyzed by HPLC for the quantification of TPA production (Fig. 2A). Minor degradation products (MHET and BHET) were also assessed (Fig. S3). We used an enzyme concentration of 40 nm (equivalent to 1.2 μg·mL^−1^ enzyme in the reaction mixture) and an amorphous PET film of 8.4 mg in a 2 mL microcentrifuge tube to evaluate the PET degradation activity of the different mutants. The enzyme concentration corresponds to ≈0.3 mg_ENZYME_/g_PET_, a ratio lower than the one reported in the original LCC‐ICCG paper (3 mg_ENZYME_/g_PET_), but in agreement with the range reported in the literature (0.2–3 mg_ENZYME_/g_PET_) [25, 26, 27, 28] The experiments were done at 68 °C, the temperature at which PET degradation is typically carried out in bioreactors at an industrial scale to work below the glass transition temperature of PET (*T*_*g*_ ≈ 70 °C). If we exclude the first few hours, where ICCG performs better, at 68 °C the mean TPA concentration at the different time points is significantly higher for the C09 mutant than for ICCG up until day 6 (144 h, Fig. 2A,B and Figs S3–S5). Moreover, the specific activity of C09 is also significantly higher (≈2‐fold) than that of the gold standard LCC‐ICCG over the same length of time (Fig. 2C). All other enzymes show similar or weaker performances compared to LCC‐ICCG (X09, C08, X05). At 68 °C, complete sample degradation could be observed with C09 already at day 4 (data not shown), a feature not seen with any of the other enzymes, including LCC‐ICCG. The percentage of weight loss after 6 days of reactions for all the enzymes is shown in Fig. 2D and in Fig. S3D. In order to assess the general functionality of the different PETase variants, we also tested the esterase activity for the PETase variants using p‐nitrophenyl acetate (pNPA) as a substrate. The specific activity against pNPA is highest for C09 and C08, followed by ICCG and X09. The mutants X05, P06, and P08 have the lowest specific activity (Fig. S6).

### Enzyme structure

**Fig. 3:**
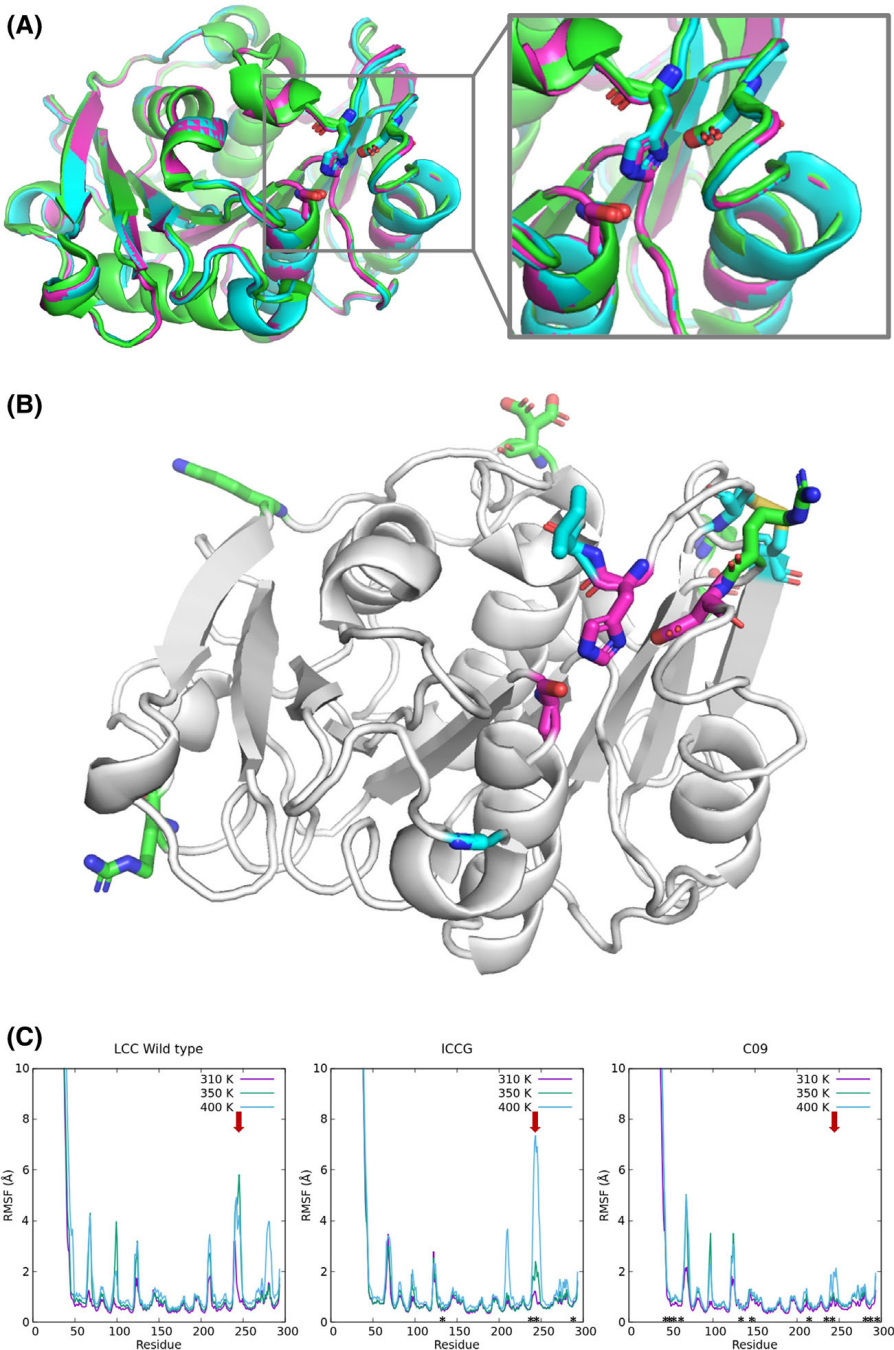
Structural comparison of relevant PETase enzymes: wild‐type LCC (green), ICCG variant (cyan), and C09 variant (pink). The overall structural alignment shows no significant differences between the enzymes, including the position and the orientation of the catalytic triad (A). Panel B shows the position of mutations (sticks) in ICCG (cyan) and C09 (pink) with respect to the catalytic site (pink sticks). Panel C shows a comparison between the Root Mean Square Fluctuation (RMSF) of LCC, LCC‐ICCG, and C09 at different temperatures (310, 350, and 400 K). The RMSF profiles show that the mutations (indicated with an asterisk) introduced in the C09 variant reduce the structural fluctuations of the enzyme, in particular near the catalytic residue H242 (red arrows). Figures in panel A and B are generated with the software PyMol [51].

We determined the crystal structure of C09 to assess whether the substitutions lead to changes in the structure of the enzyme. The structure was determined at 1.28 Å resolution. We compared the predicted secondary structure content based on CD with the experimental results, showing good alignment between the CD‐based prediction and the crystallographic structure (Table S2). The structural comparison of the C09 variant with the parent enzymes shows no significant changes in the folding, as observed by the minimal change in RMSD with respect to the WT (0.257 Å) and to the LCC‐ICCG variant (0.152 Å). The position and orientation of the catalytic triad (D210, H242, and S265) overlap perfectly with the catalytic triad in the parent enzymes (Fig. 3A). Although we observe that increased stability leads to higher enzymatic activity, a mechanistic explanation of the phenomenon is elusive. We hypothesize that the increase in stability provided by the additional surface charges in the C09 variant may help to keep the surface‐exposed catalytic site in place even at high temperatures, such as those used to test the activity of the enzyme (68 °C). Indeed, all the substitutions introduced in the design process are found mostly on the surface of the enzyme and away from the catalytic triad (Fig. 3B; Fig. S7) and are thus unlikely to affect the activity directly. Rather, the substitutions may reduce the proportion of the enzyme that may denature over time, and/or prevent the unfolding of the exposed catalytic triad. The denaturation (and the consequent loss of activity) is expected to be negligible only for *T* < *T*_*m*_ [29]. Therefore, by increasing the *T*_*m*_ to 97.1 °C, the denaturation effects may be significantly reduced at the working temperature (68 °C), thus possibly explaining the gain in activity of the C09 design. The stabilization of the catalytic triad is confirmed by MD simulations, which show that at increasing temperatures C09 features a lower RMSF, in particular near the key catalytic residue H242 (Fig. 3C).

## Discussion

Despite *Is*PETase being initially considered a promising enzyme for PET degradation, multiple studies have shown that thermophilic PET hydrolases (e.g., LCC), when operating at temperatures above 65 °C, outperform mesophilic enzymes in PET biorecycling [30]. Since its publication in 2020, the LCC‐ICCG variant has been considered the state‐of‐the‐art PETase enzyme. Hence, starting from this variant, we set out to engineer a novel enzyme with enhanced thermal stability and enzymatic activity. The resulting LCC‐ICCG‐C09 design featured a significant improvement in terms of thermal stability (*T*_*m*_ = 97.1 °C) with respect to the gold standard LCC‐ICCG (+3.5 °C). Under optimal reaction conditions (68 °C) and at laboratory scale, the LCC‐ICCG‐C09 enzyme hydrolyzed low‐crystallinity PET materials into TPA with a 2‐fold higher efficiency compared to LCC‐ICCG. The LCC‐ICCG‐C09 variant may represent a further step towards PET biorecycling. However, further work will be needed to test this enzyme in industrially relevant conditions. The LCC‐ICCG‐C09 variant could also provide the basis for further rounds of enzyme engineering.

Recent studies reported in the literature adopted similar approaches to ours, introducing substitutions to the LCC‐ICCG enzyme to boost its performance and reporting different degrees of success [26, 28, 31, 32] However, as reported in relevant reviews [33, 34] direct comparison of enzymatic performances is challenging due to the numerous variables involved in the experimental settings (PET crystallinity, PET form, PET concentration, enzyme concentration, temperature, salt type and concentration, pH, etc.). In this light, only a direct comparison with a recognized gold standard in the same experimental conditions can provide reliable information.

Although the field of PETase engineering is very dynamic and is providing highly optimized enzymes, several issues still need to be thoroughly investigated to improve enzymatic PET degradation [34]. One of the major challenges is related to the economic viability of enzymatic degradation. Another challenge, more directly related to protein engineering, concerns the acidification of the medium due to the release of TPA, which reduces the performance of the PETase enzymes known to date. Hence, possible directions for enzyme engineering research could be directed towards the design of acid‐tolerant PETase enzymes. Finally, while our study primarily compares newly developed enzymes with the current gold standard in a laboratory setting, we recognize the need for industrial‐scale validation, such as testing with post‐consumer PET in bioreactors.

## Materials and methods

### *In silico* design and selection of stabilizing mutants

We started our enzyme engineering process from the LCC‐ICCG enzyme reported by Tournier *et al*. [15]. This engineered enzyme is a mutant of the wild‐type leaf‐branch compost cutinase (LCC) carrying 4 point mutations: F243I, Y127G, S283C, and D238C. The last two substitutions are designed to induce the formation of a second disulfide bond, in addition to the native one (C275‐C292). In both enzymes, the catalytic site is centered on residue S165. The structure of the LCC‐ICCG engineered enzyme is available in the Protein Data Bank (PDB code: 6THS) as the inactivated S165A variant.

In our design strategy, we applied protocols used in previous works [17, 18, 19] starting from the LCC‐ICCG mutant structure after reverting amino acid 165 from Alanine to the catalytically active Serine. All residues within a distance of 5 Å from S165 were excluded from the design and were therefore left unchanged. The design was done using three separate methods, resulting in three different series of variants.

The first design strategy (C‐series) was performed using the Rosetta Supercharge tool [22]. This tool was employed to reengineer the protein surface with amino acids that present a high net charge, which is reported to prevent aggregation of partially unfolded states. The design produced 1024 enzyme variants, and the best 10 based on the internal score were kept for further ranking. The Rosetta Supercharge tool directly provides a 3D model in PDB format for the proposed mutants.

In the second design strategy (P‐series) we used the PROSS web server [21] to generate 9 different enzyme designs with improved packing, hydrogen bond networks, and salt bridge networks. PROSS directly provided the PDB structure of the proposed variants.

The third design strategy (X‐series) employed the Disulfide‐by‐Design web server [23] a tool for disulfide bond prediction that allows finding pairs of amino acids that can carry cysteine substitutions in the correct orientation to form disulfides. A total of 150 pair substitutions were generated and, based on the internal scoring system, the best 10 of them were retained for further testing. In this case, we used the “Mutate Residue” tool in VMD to modify the proposed positions to cysteines.

Overall, our design campaign produced 29 different enzyme variants that were then evaluated using MD simulations; then ranked according to the method described in previous works [17, 18, 19, 20] Briefly, we solvated each molecular model with a 15 Å pad of TIP3P water, and we introduced counter ions to neutralize the system charge, resulting in a final simulation box of ≈30.000 atoms. Hydrogen mass repartitioning was applied to allow a time step of 4 fs [35]. The systems were subjected to 1000 energy minimization steps, and it equilibrated for 1 ns at a pressure of 1 atm and at a temperature of 300 K with NAMD software [36] using AMBER19SB force field [37] non‐bonded cutoff of 12 Å, rigid bonds, and particle‐mesh Ewald long‐range electrostatics. During the equilibration simulation, the Cα atoms of the protein were restrained by a 10 kcal·mol^−1^·Å^−2^ spring constant. The 1 μs production runs were performed using a NVT ensemble whereby all the parameters (non‐bonded cutoff, and PME) were the same as in the equilibration phase. All simulations were run in triplicates. Root mean square deviations (RMSD) were monitored after each MD run to assess structural convergence, while root mean square fluctuations (RMSF) were calculated to assess the improved stability of the design. As a result, the two best structures from each design series were selected, namely design LCC‐ICCG‐C08, LCC‐ICCG‐C09, LCC‐ICCG‐P06, LCC‐ICCG‐P08, LCC‐ICCG‐X05, LCC‐ICCG‐X09. In the remainder of this document, the “LCC‐IGGC‐” prefix of enzyme names is omitted for brevity.

### Protein expression and purification

The different codon‐optimized nucleotide sequences of the designed PETase mutants were cloned in a pET26b(+) bacterial expression vector (Novagen, Malvern, Worcestershire, UK), together with a C‐terminal 6His‐tag. All the different mutants were transformed and expressed in *Escherichia coli* BL21 Star (DE3) cells (Invitrogen, Carlsbad, CA, USA). A sample of 4 mL of an overnight culture of the selected strain grown in the presence of 50 μg·mL^−1^ kanamycin was inoculated into 2 L of Luria–Bertani broth (LB) at 37 °C, supplemented with 50 mg·L^−1^ kanamycin and grown until OD_600_ = 0.6. The expression was induced with isopropyl 1‐thio‐β‐d‐galactopyranoside (IPTG) at a final concentration of 0.1 mm followed by overnight incubation at 18 °C and 220 rpm. Cells were collected by centrifugation and resuspended in lysis buffer (20 mm Tris/HCl pH 8.0, 300 mm NaCl) supplemented with 0.2 mm protease phenylmethylsulfonyl fluoride (PMSF) and DNAse and then lysed by sonication on ice. After centrifugation (45 000 ***g*** for 40 min at 4 °C), the soluble fraction of the cell lysate was passed through a 0.45 μm filter and then loaded onto a HisTrap HP 5 mL (GE Healthcare, Chicago, IL, USA) column equilibrated with buffer A (20 mm Tris/HCl pH 8.0, 300 mm Nacl and 10 mm imidazole). The Ni affinity column was washed using 10 column volumes of buffer A. Before elution, five column volumes (CV) of buffer A supplemented with 50 mm imidazole were used to remove unspecific proteins from the resin. Then, the bound His‐tagged enzyme was eluted with the same buffer supplemented with 500 mm imidazole in a linear gradient. The protein eluted completely with 250 mm imidazole. The eluted fraction was loaded onto a PD‐10 desalting column pre‐equilibrated with buffer C (20 mm Tris/HCl pH 8.0, 300 mm NaCl) for buffer exchange. Buffer exchange was performed simultaneously after the protein was purified. The desired protein was concentrated to ≈2 mg·mL^−1^ using an Amicon 20 centrifugal filter with a molecular cutoff of 10 kDa and stored at −80 °C. Protein concentration was assessed using a NanoDrop OneC (Thermo Fisher Scientific, Waltham, MA, USA). Sample purity was evaluated by 12% SDS/PAGE. The final yields of all the enzymes were around 16 mg·L^−1^ of bacterial culture.

### Circular dichroism

Circular dichroism (CD) measurements were performed on a Jasco J‐1500 spectropolarimeter at 20 °C. CD was performed to detect the secondary structure of different variants and to measure the thermostability of the enzyme. The spectra were determined with the following parameters: continuous scanning mode with a scanning speed of 50 nm·min^−1^, band width 0.1 nm, data pitch 0.1 nm at 20 °C. A 1.0 mm path length quartz cuvette was used for 5 μm samples of different enzymes in 20 mm Tris/HCl pH 8.0, 150 mm NaCl. Thermal denaturation curves were measured in 1.0 mm path length cuvettes closed with a parafilm on protein solutions. Protein denaturation was induced upon increasing the temperature at a rate of 1 °C·min^−1^ from 20 °C to 120 °C. The ellipticity at 222 nm was recorded at intervals of 0.2 °C using a 1‐nm bandwidth and a response of 10 s.

The determination of the midpoints of the thermal denaturation curves (*T*_*m*_) was done by fitting the data to a sigmoidal transition curve using a modified sigmoidal model [38]:

(1) $$\mathrm{CD}=\frac{\left(b\cdot T-c\right)-\left(d\cdot T-e\right)}{\left(1+{\left(\frac{T}{a}\right)}^{f}\right)+\left(d\cdot T-e\right)}$$

where *T* is the temperature, *a* represents the melting temperature (*T*_*m*_), and the terms (*b*·*T* − *c*) and (*d*·*T* − *e*) account for baseline drift at low and high temperatures, respectively. The parameter *f* controls the steepness of the transition. This model allows for accurate fitting of CD (circular dichroism) data across the full thermal range, capturing both transition and baseline behavior (Fig. S2).

The secondary structures of all the different variants were measured at 200–250 nm wavelengths and 20 °C. Ellipticity was plotted versus wavelength with GraphPad Prism 10 software. Each recorded spectrum was the average of three scans (Figs S8, S9). CD spectra for the C09 variant were further analyzed using the K2D algorithm of the DichroWeb tool [39] (Table S2).

### Enzymatic reaction

Bis(hydroxyethyl)terephthalate (BHET), mono(hydroxyethyl)terephthalate (MHET), terephthalic acid (TPA), and ethylene glycol (EG) are the major depolymerization products of the activity of the enzymes on PET. These compounds can be separated efficiently on a C18 reversed‐phase high‐performance liquid chromatography HPLC column, thus allowing a precise quantification of product formation. All enzyme reactions were performed at a concentration of 40 nm at 68 °C in quintuplicates in 2.0 mL microcentrifuge tubes in the reaction buffer (500 mm Tris/HCl pH 8.0, 300 mm NaCl). Amorphous, 250 μm‐thick PET films (product number ES301445/11) were purchased from Goodfellow USA and cut into 6 mm diameter circular pellets using a hole puncher. Individual pellets, whose weight was around 8.4 mg, were then placed in 2 mL microcentrifuge tubes. The esterase activity of enzyme variants was assessed by monitoring the hydrolysis of p‐nitrophenyl acetate (pNPA) [24]. Reactions were performed at a concentration of 0.1–1.0 μm in a 96‐well microplate at 25 °C using 1 mm pNPA in a buffer containing 20 mm Tris/HCl (pH 8.0) and 300 mm NaCl. Enzymatic activity was quantified by measuring the increase in absorbance at 405 nm (Clariostar Plus), corresponding to the formation of p‐nitrophenolate (ε₄₀₅ = 11.6 mm ^−1^·cm^−1^) (see Fig. S6).

### Reversed‐phase liquid chromatography

The different compounds produced by PET hydrolytic enzymes (TPA, as well as BHET and MHET) were quantified by reversed‐phase HPLC (Tables S3–S5). HPLC analyses were performed on a Shimadzu LC‐2030C 3D Plus system equipped with a PDA detector, a column oven, and an autosampler (Shimadzu, Kyoto, Japan). Separations were carried out using a Kinetex C18 column (2.7 μm; 4.6 × 150 mm, Phenomenex, Torrance, CA, USA). Column temperature was maintained at 40 °C with a flow rate of 1 mL·min^−1^. For the mobile phase, 0.1% H_3_PO_4_ (solvent A) and MeCN (solvent B) were used. The linear gradient mode used for the elution was as follows: 0 min, 10% B; 0–15 min, 10–40% B; 15–18 min, 40–65% B; 18–20 min, 65% B; 20–23 min, 65–10% B; 23–25 min, 10% B. At every time interval, 100 μL of the sample was collected from the reaction vial placed at the set temperature. The 0–72 h samples were diluted 21 times (50 μL of the provided sample was transferred to autosampler vial and diluted in 1 mL of 10% MeCN +0.1% H_3_PO_4_), whereas the 96 h and 144 h samples were diluted 101 times (10 μL of the provided sample was transferred to autosampler vial and diluted in 1 mL of 10% MeCN +0.1% H_3_PO_4_). Detection was accomplished via measurement of UV adsorption at 240 nm; 20 μL of each sample was injected into the system, and the degradation products were quantified against standard calibration curves ranged 0.1, 1.0, 5.0, 10.0, 50.0 (for TPA and MHET) and 0.09, 0.9, 4.4, and 43.9 (for BHET) μg·mL^−1^.

### Protein crystallization

Crystals of the C09 mutant were grown at room temperature using the vapor diffusion method by mixing a 2 μL drop of a 10 mg·mL^−1^ protein sample with a 2 μL drop of a solution containing 0.1 m Sodium citrate tribasic dihydrate pH 5.6, 20% v/v 2‐Propanol, 20% w/v Polyethylene glycol 4000. Crystals, which appeared after one week, were frozen in liquid nitrogen using 25% (v/v) glycerol as a cryoprotectant prior to X‐ray diffraction data collection.

### Structure determination and refinement

For the structure of the C09 mutant, the initial phases were obtained by molecular replacement using Phaser [40] and the atomic coordinates of the crystal structure of a Leaf‐branch compost bacterial cutinase homolog (PDB entry 4EB0) as a search model. Refinement was performed by alternating rounds of REFMAC5 [41] and manual adjustments in Coot [42] Water molecules were added both manually and automatically using the Coot_refine tool from the CCP4 package [43] The crystallographic table is shown in the SI file in Table S6.

## Conflict of interest

The authors declare no conflict of interest.

## Author contributions

SB, RC contributed to methodology, investigation, formal analysis, writing, and editing of the manuscript. HE, TU, AR contributed to investigation. AG, EP conceptualized and designed the research, contributed to formal analysis, writing, and editing of the manuscript. All authors reviewed and approved the final version of the manuscript.

## Supporting information


**Fig. S1.** Sequence alignment.
**Fig. S2.** Representative melting curves.
**Fig. S3.** Product formation and PET film degradation.
**Fig. S4.** Time course of the terephthalic acid (TPA) production from different enzymes.
**Fig. S5.** Terephthalic acid (TPA) production from different enzymes.
**Fig. S6.** Comparison of the specific activity of each enzyme variant against p‐Nitrophenyl Acetate (pNPA) and Polyethylene terephthalate (PET).
**Fig. S7.** Electrostatic potential surfaces, calculated by the Adaptive Poisson‐Boltzmann Solver module as implemented in PyMol for ICCG (A) and C09 (B).
**Fig. S8.** Secondary structure measurements by circular dichroism.
**Fig. S9.** Secondary structure measurements by circular dichroism.
**Table S1.** Production yields of the recombinant enzyme variants.
**Table S2.** Experimental and predicted secondary structures for the C09 enzyme.
**Table S3.** Terephthalic Acid (TPA) production as measured by high‐performance liquid chromatography.
**Table S4.** Mono(hydroxyethyl)terephthalate (MHET) production as measured by high‐performance liquid Chromatography.
**Table S5.** Bis(hydroxyethyl)terephthalate (BHET) production as measured by high‐performance liquid chromatography.
**Table S6.** Diffraction data collection and refinement statistics.

## Data Availability

All data generated or analyzed during this study are included in this published article and its Supporting Information file. The final crystallographic coordinates of the crystal structure of the C09 mutant (accession code: 8CMV) are available in the RCSB PDB (https://www.rcsb.org).
