# Supplementary material for: Development of a highly active engineered PETase enzyme for polyester degradation
Source: FEBS J. 2025 Aug 23;293(2):443–55. doi: 10.1111/febs.70228 (PMC12820602; doi:10.1111/febs.70228)
Supplement: Supplementary file 1 — Fig. S1. Sequence alignment. Fig. S2. Representative melting curves. Fig. S3. Product formation and PET film degradation. Fig. S4. Time course of the terephthalic acid (TPA) production from different enzymes. Fig. S5. Terephthalic acid (TPA) production from different enzymes. Fig. S6. Comparison of the specific activity of each enzyme variant against p‐Nitrophenyl Acetate (pNPA) and Polyethylene terephthalate (PET). Fig. S7. Electrostatic potential surfaces, calculated by the Adaptive Poisson‐Boltzmann Solver module as implemented in PyMol for ICCG (A) and C09 (B). Fig. S8. Secondary structure measurements by circular dichroism. Fig. S9. Secondary structure measurements by circular dichroism. Table S1. Production yields of the recombinant enzyme variants. Table S2. Experimental and predicted secondary structures for the C09 enzyme. Table S3. Terephthalic Acid (TPA) production as measured by high‐performance liquid chromatography. Table S4. Mono(hydroxyethyl)terephthalate (MHET) production as measured by high‐performance liquid Chromatography. Table S5. Bis(hydroxyethyl)terephthalate (BHET) production as measured by high‐performance liquid chromatography. Table S6. Diffraction data collection and refinement statistics. [file FEBS-293-443-s001.pdf]

Supplementary Data

Development of a highly active engineered PETase enzyme for polyester degradation

Shapla Bhattacharya[a],[b]≠, Rossella Castagna[a],[c]≠, Hajar Estiri[a],≠, Toms Upmanis[a],[d], Andrea Ricci[e], Alfonso Gautieri*[e], Emilio Parisini*[a],[f]

[a] Department of Biotechnology, Latvian Institute of Organic Synthesis, Aizkraukles 21, LV-1006, Riga, Latvia
[b] Institute of Chemistry and Chemical Technology,Faculty of Natural Sciences and Technology, Riga Technical University, Paula Valdena 3, LV-1048, Riga, Latvia
[c] Dipartimento di Chimica, Materiali e Ingegneria Chimica "G. Natta", Politecnico di Milano, Piazza Leonardo da Vinci 32, 20133 Milano, Italy
[d] Faculty of Chemistry, University of Latvia, Jelgavas iela 1, LV-1004 Riga, Latvia
[e] Biomolecular Engineering Lab, Dipartimento di Elettronica, Informazione e Bioingegneria, Politecnico di Milano, Piazza Leonardo da Vinci 32, 20133 Milano, Italy
[f] Department of Chemistry "G. Ciamician", University of Bologna, Via P. Gobetti 85, 40129 Bologna, Italy

≠ These three authors contributed equally to this work

* To whom correspondence should be addressed: Alfonso Gautieri (alfonso.gautieri@polimi.it), Emilio Parisini (emilio.parisini@osi.lv)

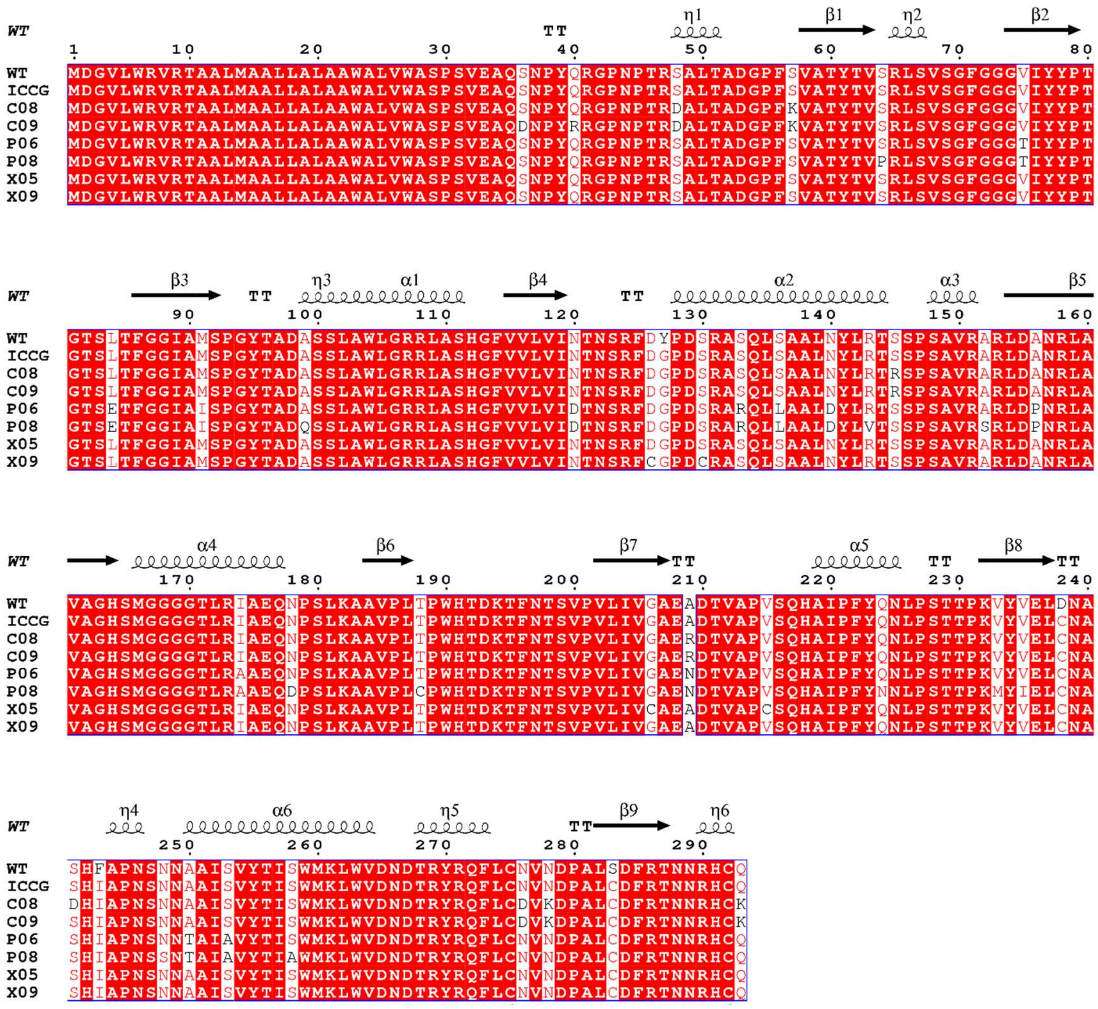

Figure S1. Sequence alignment. Sequence alignment of the wild type LCC enzyme (Uniprot ID G9BY57), the ICCG variant developed by Tournier et al., and the engineered enzymes developed in this work (C08, C09, P06, P08, X05, X09). Alignment is performed with Clustal Omega, while the graphical representation is obtained using ESPrit 3 web server.

Circular dichroism curves corresponding to the denaturation temperature (T_m) for the different mutants, measured from 20 °C to 120 °C at 222 nm.

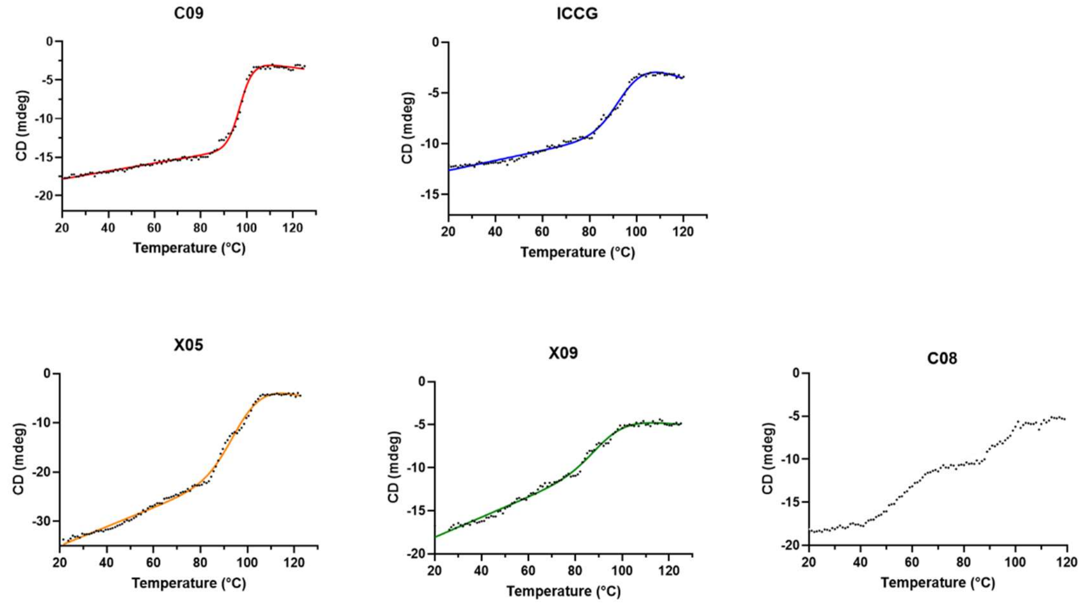

Figure S2. Representative melting curves. Each panel show a representative melting curve measured by circular dichroism at 222 nm from 20°C to 120°C for the corresponding sample; black dots represent experimental data points, and the solid line indicates the fitted curve. For sample C08, it was not possible to obtain a reliable fit.

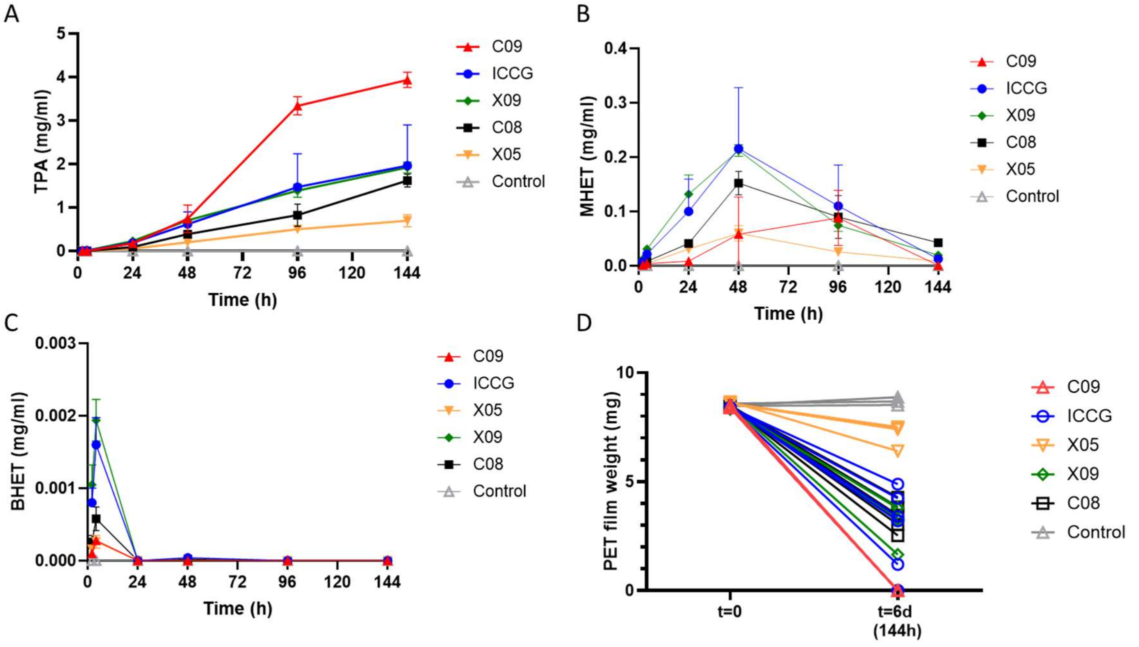

Figure S3. Product formation and PET film degradation. A) Terephthalic acid *(*TPA) production over time quantified by High Performance Liquid Chromatography (HPLC) analysis after reaction with 40 nM enzyme (ICCG – C09 – X05 – X09 – C08 (n=5) – no enzyme (control n=3)). The error bars denote standard deviation. Data points have been reused from Figure 2A B) Bis(2-hydroxyethyl) terephthalate (BHET) production over time quantified by HPLC analysis after reaction with 40 nM enzyme (ICCG – C09 – X05 – X09 – C08 (n=5) – no enzyme (control n=3)). The error bars denote standard deviation. C) MHET production over time quantified by HPLC analysis after reaction with 40 nM enzyme (ICCG – C09 – X05 – X09 – C08 (n=5) – no enzyme (control n=3)). The error bars denote standard deviation. D) PET film weight before (t=0) and after treatment (t=6d, 144h) with 40 nM enzyme (ICCG – C09 – X05 – X09 – C08 (n=5), no enzyme (control n=3)). Mean weight loss for ICCG at 6 days is 61%. Mean weight loss for C09 at 6 days is 100%.

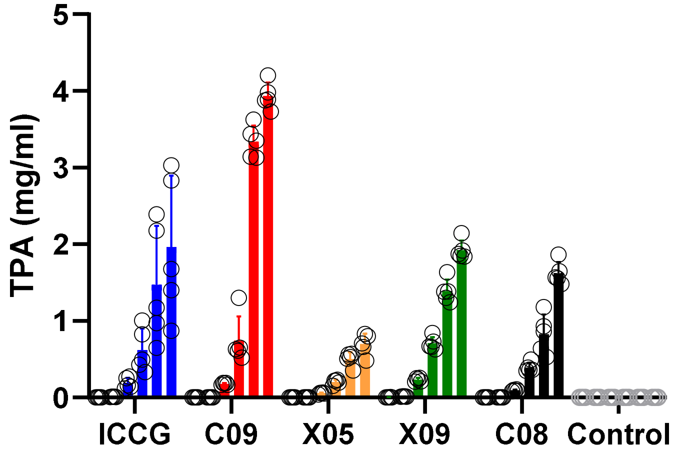

Figure S4. **Time course of the terephthalic acid (TPA) production from different enzymes.** Experiments performed using 40 nM enzyme at 68°C (n=5). Time points: 2h, 4h, 24h, 48h, 96h and 144h. The error bars denote standard deviation. Data points have been reused from Figure 2A.

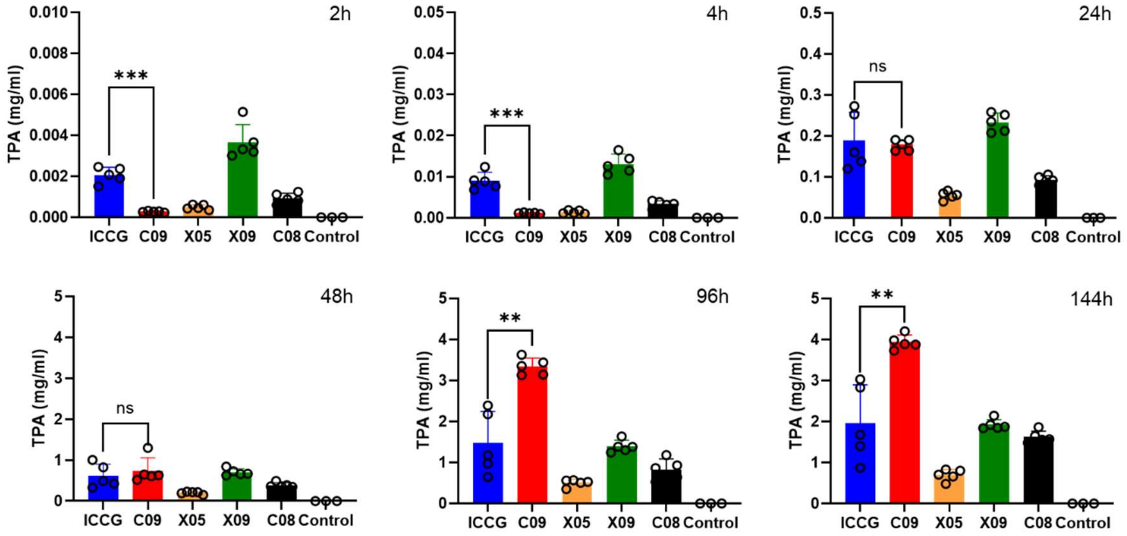

Figure S5. **Terephthalic acid (TPA) production from different enzymes** (ICCG – C09 – X05 – X09 – C08 (n=5)) and control (no enzyme (n=3)) at 40 nM, 68°C and at different time points (2h, 4h, 24h, 48h, 96h and 144h). ns (non significant); **P<0.01; ***P<0.001 (one-tailed unpaired Welch's t-test). The error bars denote standard deviation. Data points have been reused from Figure 2A and Figure 2B.

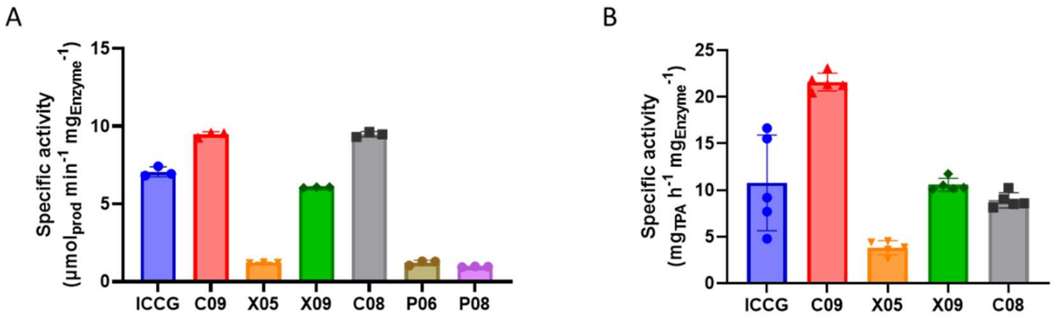

Figure S6. **Comparison of the specific activity of each enzyme variant against p-Nitrophenyl Acetate (pNPA) and polyethylene terephthalate (PET).** A) Esterase activity against pNPA was assessed measuring the absorbance at 405 nm, corresponding to the formation of p-nitrophenolate (prod) at the endpoint of 10.55 min. Reactions were performed in triplicates (n=3) in a 96-well microplate at 25°C using 1 mM pNPA in a buffer containing 20 mM Tris-HCl (pH 8.0) and 300 mM NaCl with enzyme concentration in the range of 0.6-0.07 µg/ml (ICCG = 0.12 µM; C09 = 0.12 µM ; X05 = 1.06 µM; X09 = 0.2 µM; C08 = 0.11 µM; P06 = 1.04 µM; P08 = 0.90 µM). The specific activity was measured as the ratio of µmol of product produced over time and the mg of enzyme used in the reaction. The error bars denote standard deviation. B) PET hydrolysing specific activity against amorphous PET films. All enzyme reactions were performed at a concentration of 40 nM at 68°C in quintuplicates (n=5) in 2.0 mL microcentrifuge tubes in the same reaction buffer (20 mM Tris-HCl pH 8.0, 300 mM NaCl). Amorphous PET films (6 mm circular disks of 8.4 mg weight) were then placed in 2 ml microcentrifuge tubes. The specific activity was measured as the ratio of mg of terephthalic acid (TPA) product produced (measured by High-Performance Liquid Chromatography) over time (endpoint of 144 h) and the mg of enzyme used in the reaction. The error bars denote standard deviation. Data points at 144h have been reused from Figure 2C.

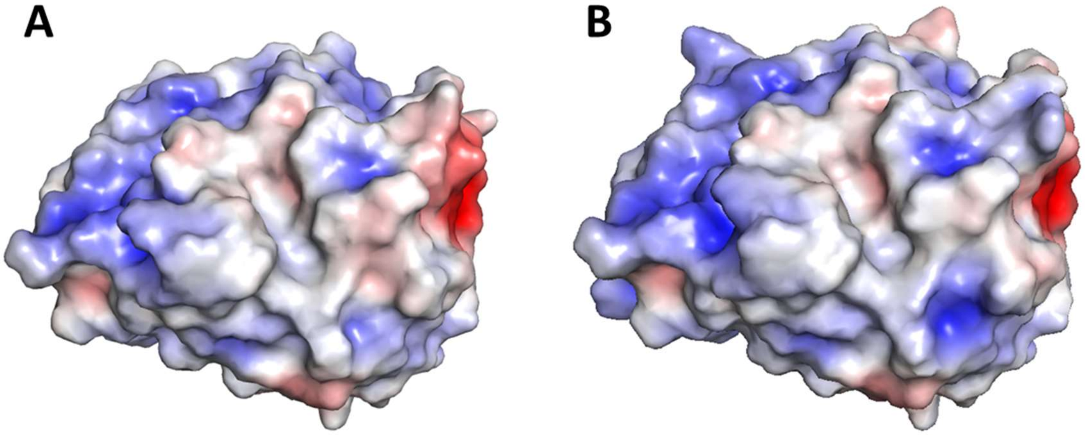

Figure S7. Electrostatic potential surfaces, calculated by the Adaptive Poisson-Boltzmann Solver module as implemented in PyMol for ICCG (A) and C09 (B). Red spheres indicate negative charges, while blue spheres represent positive charges. The positively and negatively charged surfaces are colored blue and red, respectively, and the electro-neutral (or nonpolar/hydrophobic) surface is shown in white. The catalytic cleft is shown at the center of the two images. C09 features a more positively charged surface than ICCG. Figures are generated with PyMol.

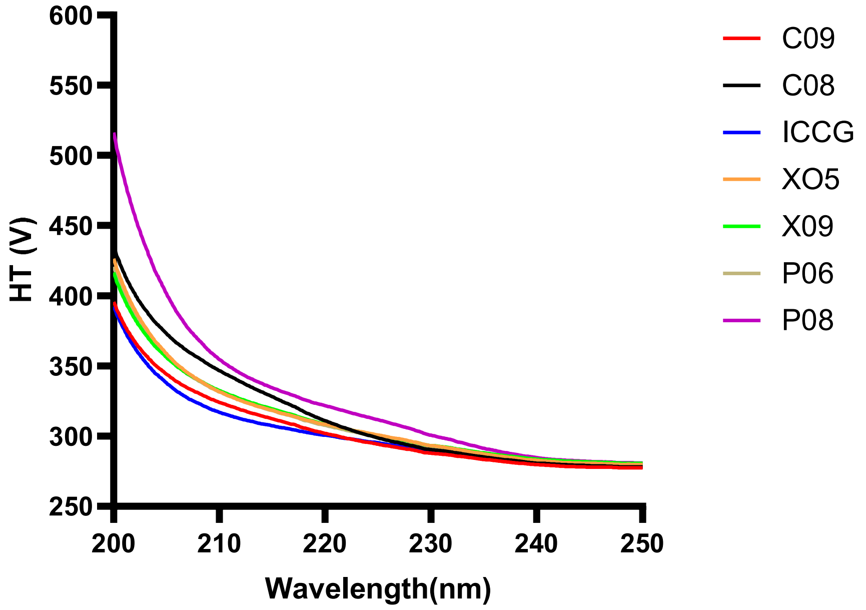

Figure S8. Secondary structure measurements by circular dichroism. The plot shows voltage vs. wavelength for the different enzymes.

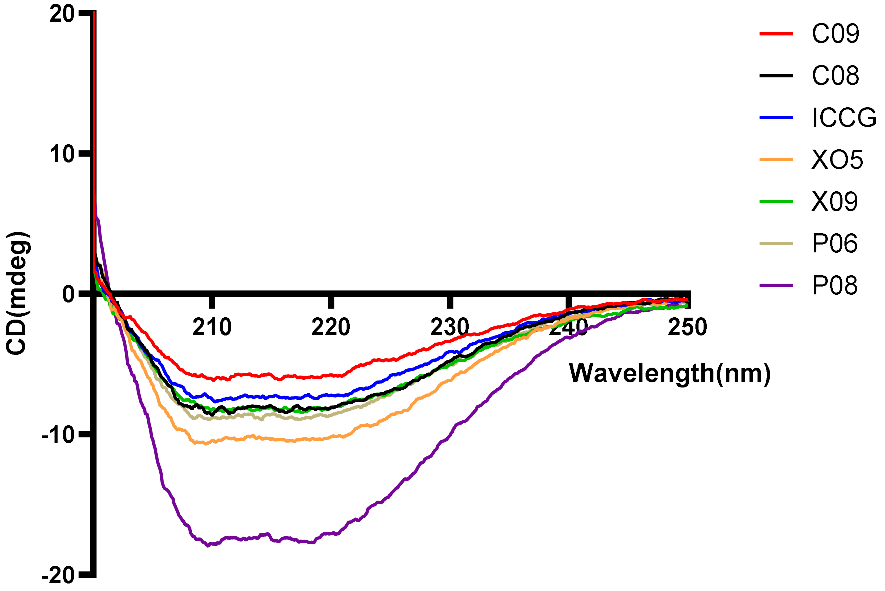

Figure S9. Secondary structure measurements by circular dichroism. The plot shows ellipticity vs. wavelength for the different enzymes.

Table S1. Production yields of the recombinant enzyme variants.

Enzyme	Production yield (mg/L)
C09	18
X05	20
X09	22
C08	18
P06	12
P08	15
ICCG	20

Table S2. Experimental and predicted secondary structures for the C09 enzyme.

**Secondary Structures**	**Crystal structure (8CMV)**	**Circular Dichroism (Dichroweb)**
Helices	0.38	0.34
Sheets	0.21	0.20
Loops	0.40	0.47

Table S3. Terephthalic acid (TPA) production as measured by High-Performance Liquid Chromatography. Quantification of TPA (mg/mL) in reaction supernatants represented in Figure 2A. Reactions were carried out using 40 nM enzyme at 68°C.

Enzyme (replicate #)	Time, h 2	4	24	48	96	144
**C09(1)**	0.00023	0.0009	0.16	0.52	3.14	3.73
**C09(2)**	0.00028	0.0011	0.16	1.30	3.63	3.88
**C09(3)**	0.00028	0.0011	0.18	0.65	3.35	4.20
**C09(4)**	0.00032	0.0013	0.19	0.63	3.44	3.98
**C09(5)**	0.00026	0.0012	0.19	0.61	3.13	3.89
**X05(1)**	0.00043	0.0011	0.060	0.23	0.57	0.83
**X05(2)**	0.00062	0.0019	0.056	0.22	0.56	0.80
**X05(3)**	0.00046	0.0012	0.052	0.20	0.50	0.66
**X05(4)**	0.00061	0.0018	0.066	0.22	0.53	0.71
**X05(5)**	0.00035	0.0010	0.040	0.15	0.35	0.48
**X09(1)**	0.0033	0.0125	0.26	0.84	1.64	2.14
**X09(2)**	0.0032	0.0115	0.21	0.66	1.38	1.84
**X09(3)**	0.0051	0.0165	0.25	0.73	1.24	1.92
**X09(4)**	0.0030	0.0105	0.21	0.63	1.30	1.85
**X09(5)**	0.0037	0.0144	0.23	0.67	1.38	1.87
**ICCG(1)**	0.0025	0.0124	0.25	0.83	2.18	2.83
**ICCG(2)**	0.0021	0.0091	0.27	1.01	2.39	3.03
**ICCG(3)**	0.0024	0.0089	0.16	0.50	1.17	1.68
**ICCG(4)**	0.0019	0.0077	0.12	0.33	0.65	0.87
**ICCG(5)**	0.0015	0.0068	0.14	0.43	0.97	1.40
**C08(1)**	0.0009	0.0020	0.079	0.35	1.18	1.87
**C08(2)**	0.0008	0.0034	0.084	0.36	0.93	1.57
**C08(3)**	0.0012	0.0042	0.091	0.50	0.86	1.48
**C08(4)**	0.0011	0.0039	0.106	0.39	0.64	1.65
**C08(5)**	0.0006	0.0032	0.099	0.36	0.53	1.56
**Control(1)**	< LOD	<LOD	< LOD	<LOD	< LOD	<LOD
**Control(2)**	< LOD	<LOD	< LOD	<LOD	< LOD	<LOD
**Control(3)**	< LOD	<LOD	< LOD	<LOD	< LOD	<LOD

< LOQ - below the lowest analyte concentration that can be quantitatively detected with a stated accuracy and precision;
< LOD - no peak found

Table S4. Mono(hydroxyethyl)terephthalate (MHET) production as measured by High-Performance Liquid Chromatography. Quantification of MHET (mg/mL) in reaction supernatants. Reactions were carried out using 40 nM enzyme at 68°C.

Enzyme	Time, h					
(replicate #)	2	4	24	48	96	144
C09(1)	0.0011	0.0032	0.008	0.02	0.14	0.0005
C09(2)	0.0013	0.0037	0.008	0.18	0.01	0.0004
C09(3)	0.0014	0.0035	0.010	0.03	0.07	0.0003
C09(4)	0.0016	0.0041	0.009	0.03	0.12	0.0007
C09(5)	0.0014	0.0039	0.007	0.03	0.10	0.0024
X05(1)	0.0015	0.0034	0.035	0.08	0.033	0.011
X05(2)	0.0025	0.0055	0.030	0.06	0.029	0.010
X05(3)	0.0017	0.0034	0.032	0.06	0.026	0.008
X05(4)	0.0026	0.0056	0.035	0.06	0.026	0.008
X05(5)	0.0015	0.0034	0.022	0.04	0.014	0.005
X09(1)	0.011	0.030	0.19	0.22	0.093	0.023
X09(2)	0.011	0.028	0.11	0.22	0.074	0.019
X09(3)	0.017	0.037	0.14	0.22	0.063	0.018
X09(4)	0.010	0.025	0.10	0.20	0.073	0.020
X09(5)	0.012	0.034	0.12	0.20	0.070	0.017
ICCG(1)	0.0076	0.028	0.17	0.30	0.18	0.0101
ICCG(2)	0.0087	0.019	0.16	0.36	0.20	0.0082
ICCG(3)	0.0090	0.022	0.06	0.19	0.08	0.0203
ICCG(4)	0.0054	0.020	0.05	0.09	0.03	0.0063
ICCG(5)	0.0066	0.019	0.06	0.14	0.06	0.0184
C08(1)	0.0019	0.0050	0.044	0.14	0.15	0.0472
C08(2)	0.0037	0.0087	0.034	0.15	0.10	0.0429
C08(3)	0.0041	0.0101	0.039	0.19	0.09	0.0388
C08(4)	0.0029	0.0097	0.046	0.14	0.06	0.0434
C08(5)	0.0031	0.0081	0.043	0.14	0.05	0.0401
Control(1)	< LOD	<LOD	< LOD	<LOD	< LOD	<LOD
Control(2)	< LOD	<LOD	< LOD	<LOD	< LOD	<LOD
Control(3)	< LOD	<LOD	< LOD	<LOD	< LOD	<LOD

< LOQ - below the lowest analyte concentration that can be quantitatively detected with a stated accuracy and precision;
< LOD - no peak found

Table S5. Bis(hydroxyethyl)terephthalate (BHET) production as measured by High-Performance Liquid Chromatography. Quantification of BHET (mg/mL) in reaction supernatants. Reactions were carried out using 40 nM enzyme at 68°C.

Enzyme	Time, h					
(replicate #)	2	4	24	48	96	144
C09(1)	0.0001	0.0002	<LOD	<LOD	<LOQ	<LOD
C09(2)	0.0001	0.0003	<LOD	<LOQ	<LOD	<LOD
C09(3)	0.0001	0.0003	<LOD	<LOD	<LOD	<LOD
C09(4)	0.0001	0.0003	<LOD	<LOD	<LOD	<LOD
C09(5)	0.0001	0.0003	<LOD	<LOD	<LOD	<LOD
X05(1)	0.00011	0.0002	<LOQ	<LOQ	<LOQ	<LOQ
X05(2)	0.00020	0.0003	<LOQ	<LOQ	<LOQ	<LOD
X05(3)	0.00014	0.0002	<LOQ	<LOQ	<LOQ	<LOQ
X05(4)	0.00022	0.0004	<LOQ	<LOQ	<LOQ	<LOQ
X05(5)	0.00012	0.0002	<LOQ	<LOQ	<LOQ	<LOD
X09(1)	0.00092	0.0021	<LOQ	<LOQ	<LOQ	<LOD
X09(2)	0.00091	0.0018	<LOQ	<LOQ	<LOQ	<LOQ
X09(3)	0.00151	0.0022	<LOQ	<LOQ	<LOQ	<LOQ
X09(4)	0.00083	0.0015	<LOQ	<LOQ	<LOQ	<LOD
X09(5)	0.00107	0.0021	<LOQ	<LOQ	<LOQ	<LOD
ICCG(1)	0.00077	0.0020	<LOQ	0.0001	<LOQ	<LOD
ICCG(2)	0.00100	0.0010	<LOQ	<LOQ	<LOQ	<LOD
ICCG(3)	0.00096	0.0018	<LOQ	0.0001	<LOQ	<LOQ
ICCG(4)	0.00049	0.0016	<LOQ	<LOQ	<LOQ	<LOD
ICCG(5)	0.00078	0.0016	<LOQ	<LOQ	<LOQ	<LOD
C08(1)	0.00012	0.0003	<LOQ	<LOQ	<LOQ	<LOQ
C08(2)	0.00033	0.0006	<LOQ	<LOQ	<LOQ	<LOQ
C08(3)	0.00035	0.0007	<LOQ	0.0001	<LOQ	<LOQ
C08(4)	0.00024	0.0007	<LOQ	<LOQ	<LOQ	<LOQ
C08(5)	0.00025	0.0006	<LOQ	<LOQ	<LOQ	<LOQ
Control(1)	< LOD	<LOD	< LOD	<LOD	< LOD	<LOD
Control(2)	< LOD	<LOD	< LOD	<LOD	< LOD	<LOD
Control(3)	< LOD	<LOD	< LOD	<LOD	< LOD	<LOD

< LOQ - below the lowest analyte concentration that can be quantitatively detected with a stated accuracy and precision;
< LOD - no peak found

Table S6. **Diffraction data collection and refinement statistics.** Statistics for the highest-resolution shell are shown in parentheses.

Structure	LCC-ICCG-C09
PDB code	8CMV
Wavelength (Å)	0.7338
Space group	$P6_3$
Unit cell axes (Å)	a=108.876, b=108.876, c=35.402,
Unit cell angles (°)	α =90.00, β =90.00, γ =120.00
Resolution range (Å)	54.497-1.280 (1.302-1.280)
Total reflections	1293458(62613)
Unique reflections	62178(3088)
Multiplicity	20.8(20.3)
Completeness (%)	100.0 (100.0)
mean(I) / sig(I)	6.5 (0.3)
Wilson B-factor	15.27
Rmerge	0.294 (13.691)
Rmeas	0.302 (14.041)
Rpim	0.066 (3.103)
CChalf	0.998 (0.346)
Reflections in refinement	62017 (6053)
Reflections in free set	3153 (317)
Rwork	0.1634
Rfree	0.1871
RMSD bonds (Å)	0.012
RMSD angles (°)	1.77
Ramachandran favoured (%)	98.44
Ramachandran allowed (%)	1.56
Ramachandran outliers (%)	0
Rotamer outliers (%)	0.9
Clash score	5.65
Overall number of atoms (non-H)	2286
in macromolecules	2010
in ligands	28
in solvent	248
Average B-factor ($Å^2$)	16.14
for macromolecules	14.3
for ligands	37.33
for solvent	28.7
